# Two Cases of Placenta Prævia

**Published:** 1859-09

**Authors:** S. F. Starley

**Affiliations:** Springfield, Texas


ART. IV.—*Two Cases of Placenta Prævia.* By S. F. STARLEY, M.D., of Springfield, Texas.

THERE is, perhaps, no subject within the whole range of obstetric science that should be considered of more importance than placenta prævia. Important, because in almost every instance the life of the mother or child depends upon the skill and judgment of the accoucheur, and the decision and promptness with which he acts. Believing it to be the duty of every one engaged in the practice of medicine, surgery, or obstetrics, to record such of his experience as may add to the mass of facts from which useful and practical deductions may be drawn, I offer the following cases to the medical profession, hoping thereby to add my mite to the facts already brought to light in connection with the interesting subject to which they relate. The first case was more immediately under the care of my partner, Dr. John L. Alston, by whom the following report was drawn up. The second occurred under my own care, though we were both concerned in the management of each.

CASE I.—I was summoned, on the twenty-first of February last, to attend Mrs. P., twenty-five years of age, in the absence of her family physician. She thought that she was about eight and a half months advanced in her seventh pregnancy, but could not tell exactly, as she had not menstruated since her last abortion. She is a stout and healthy-looking woman, and says that she has always enjoyed good health during all her pregnancies except the last; had brought three children, at their full terms, with quick and easy labors, and had had two abortions from slight accidents, as she supposed. On questioning her, I ascertained that she had never had leucorrhœa, or any other symptom of inflammation of the os uteri, either during or after pregnancy; that she had had slight hemorrhage every day or two for the last four or five weeks, and once or twice had lost so much blood that she became alarmed, and sent for her family physician. On the day that I was consulted, profuse hemorrhage occurred, but had ceased before my arrival. From this attack she must have lost a considerable quantity of blood, judging from the clots I found about the bed, and her prostrated condition. She had not felt any pain in the back, or any uterine contractions during this or the preceding bleeding. Pulse, 120 per minute, and feeble. Complained of roaring in the head and intolerance of noise; faintness on rising up; face blanched; great thirst; irritable stomach, etc. I told her that from the absence of symptoms of uterine disease and pains during the hemorrhages, I apprehended a placenta prævia, but would postpone a vaginal examination until the bleeding should recur, fearing lest in making the examination I would remove the coagulum formed, and renew the bleeding. I gave her opium and acetate of lead to quiet the nervous and vascular systems, and to act as an astringent; and, as there was no hemorrhage going on, I provided myself with a quantity of clean, raw cotton, to make a tampon if it should be required. I ordered the medicine to be given every two hours, and laid down to sleep, with express command to the nurse that I should be awakened if hemorrhage should again take place. This did not occur, however, and on the morning of the next day I found her much better in every respect; but about ten o'clock A.M., the bleeding commenced again profusely. I then made an examination per vaginam, and found the os uteri high up and far back toward the promontory of the sacrum, and slightly dilated. By patient effort I passed my finger through the os, and ascertained that the placenta covered the opening. I immediately withdrew my finger and introduced the tampon, rolling the cotton into a cylinder, as directed by Professor Henry Miller, in his System of Obstetrics, and securing it with a T-bandage. I packed the vagina so full of cotton that the upper portion of the canal was stretched, and formed a cone, with the apex at the vulva. She went on well during the day, but toward evening labor-pains set in, and increased during the night. About midnight a hemorrhage made its appearance at the vulva, and on examining, I found the tampon loose. I did not, however, withdraw the latter, but pressed it up as high as I could, with due regard to the parts, and filled the vagina below full of fresh cotton. This arrested the bleeding effectually.

On the twenty-third, the pains became stronger and more rapid; I sent to town for my partner to assist me. The pains soon became so violent that I concluded to remove the tampon to ascertain the condition of things, and, on making an examination, found the os uteri dilated to the diameter of an inch and a half, and the vertex presenting. The hemorrhage had ceased, and the membranes were protruding; the placenta was adhering to the front and right side of the cervix, and had merely reached across the os to the left side before the latter commenced dilating. The os dilated slowly, and a great many pains made but little impression on it. I now considered the case as though the placenta had grown to the upper part of the uterus, and treated it accordingly. To increase the contractions I used orificial stimulation by pressure against the front and right side of the os uteri during the pains. At eleven o'clock A.M. she was taken with a convulsion, after which I gave her some whisky, and just enough chloroform, by inhalation, to stimulate the nervous system, keeping her head very low all the time. She soon had another convulsion. By this time my partner had arrived. We then concluded to give her whisky and morphine; by persevering in the use of which she had no more spasms. Labor now progressed favorably, and as soon as the head and shoulders passed the vulva, in order to cause the uterus to contract firmly, while supporting the perinæum with my right hand, I immersed my left into a pan of cold water, and applied it firmly over the lower part of the abdomen. As soon as the hips passed, my partner dipped both his hands into the water and made pressure in the same manner. As soon as we could we gave her half a drachm of oil of ergot. The placenta was thrown off without the loss of any blood. The child, a boy of average size, was still-born. We gave it up as lost only after having used the Marshall Hall method of resuscitation for an hour; the mother made a speedy recovery.

CASE II.—On the evening of the twenty-fourth instant I was called to attend Mrs. W., aged about twenty-eight years, in the eighth and a half month of her fifth pregnancy. At six o'clock P.M. she was seized with labor-pains, attended with profuse hemorrhage. Owing to her residence being four miles from town, and her husband having some difficulty in sending for me, I did not reach her residence until five hours had elapsed from the time the pain and flooding commenced. On my arrival, the husband informed me that she had had some hemorrhage, every few days, for about four weeks. I found her blanched and exhausted from the loss of blood; the pains had nearly ceased; the uterus was lax and non-contractile; pulse small and frequent; great restlessness; excessive thirst, and the stomach so irritable that the water, which was incessantly called for, was rejected almost as soon as swallowed. On an examination per vaginam, I found the os uteri dilated to the size of a dollar, with a complete placental presentation, the vertex being the presenting portion of the fœtus. The blood was flowing rapidly, and the patient began to faint while I was making the examination. I had the head lowered instantly, and administered half a grain of morphine in some whisky, which had the effect of somewhat restoring her. I then attempted to detach that portion of the placenta which was within reach of my finger, after the plan proposed by Dr. Robert Barnes in his Lettsomian Lectures, (May, 1857;) but the hemorrhage was so profuse, the uterus so perfectly lax, the manipulations not exciting the slightest contraction, and the patient apparently sinking rapidly, I desisted, and resorted to plugging, which I did with all possible haste. The material used for this purpose was cotton, care being taken to pack it firmly against the os uteri, so that the vagina was tightly fitted quite down to the vulva. A towel was then folded and rolled into a cylinder about nine inches in length, which was rolled lengthwise in another towel, thus forming a cylindrical pad, which was applied to the vulva, one end of the long towel being brought up behind and the other in front, and both fastened to a handkerchief passed over the shoulder, in the form of a suspender. In this way firm pressure was made against the perinæum, and the plug more firmly maintained in situ. The hemorrhage now seemed to be arrested; but the quantity of blood already lost was so great, there being at least half a gallon on the bed, that it required the free exhibition of brandy and opiates to support life. My partner, Dr. Alston, having arrived, it was agreed that we should endeavor to sustain the patient by stimulants, nourishment, and opiates, until uterine contractions should again set up, which we believed the plug would call into action. But in this we were mistaken. She continued restless, with hurried breathing and occasional vomiting, until two o'clock on the twenty-fifth, when, after a fit of retching, I found that blood was escaping, notwithstanding the plug had been tightly packed in the vagina. On passing the finger I found the plug lying loose, which I supposed was owing to the relaxation and yielding of the vaginal walls. I removed the tampon, and finished the operation of detaching as much of the placenta as remained within reach of the finger. The uterus would not contract. The blood continued to flow while I was manipulating the os. The vagina was immediately replugged, so that no more blood could escape. We gave ergot, injected cold water into the rectum, applied cloths wrung out of cold water to the abdomen and vulva, used friction over the abdominal muscles, and gave as much brandy as we could get her to swallow, but all to no effect. She gradually sank, and died at seven o'clock P.M., no contractions having taken place after I saw her. Some slight pains were complained of, but if the uterus contracted at all, its action was too feeble to be felt through the abdominal parietes.

In this case labor had evidently begun, for the husband informed me that he expected the delivery would take place before I could reach her, so violent were the pains at the onset. But six hours having elapsed before my arrival, she had lost enough blood to prostrate her and suspend the action of the uterus. I am aware that the treatment pursued in the case is open to the criticism of those whose only anchor of hope is chained to the operation of turning and forced delivery, and perhaps I shall not escape the censure of those who can see no safety except in the total detachment of the placenta after the manner recommended by Professor Simpson. But had artificial delivery been attempted, it is evident to my mind that she would have died before the uterus could have been emptied. And granting that she could have survived long enough for the completion of the operation, is there any reason to believe that the uterus would have contracted and put a stop to the fatal drain? On the other hand, had I effected the total detachment of the placenta, would that have staunched the flow of blood? I think not. What then could I do? The orificial irritation consequent upon the separation of a portion of the placenta failed to excite the least contraction. The flood-gates seemed to be lifted still higher by it. What else could I resort to but the tampon? It is agreed by all authorities that the plug is an efficient agent in bringing on uterine contractions. Professor Henry Miller relies exclusively on it to excite contractions, and to control the flooding until the liquor amnii is evacuated. He says this is the only method of treatment of which he has any experience, and he has employed it with uniform success, so far as the mother is concerned.

The operation proposed by Dr. Barnes is to carry the detachment farther, so as to separate all that portion of the placenta which adheres within the cervical zone or region of dangerous placental seat. Speaking of the cases in which this operation may be expected to succeed, he says: "There are cases in which contractions of the uterus are going on; there are cases more dangerous still, in which contraction is absent. Labor with relaxation is dangerous under almost every condition; it is eminently so when complicated with placenta prævia. Where contraction is present we possess one necessary element of safety; we may be satisfied with the artificial separation of the placenta from its attachment to the cervical zone. Where contraction is absent, we must at the same time use every available means of rousing the contractile energy of the womb. Sometimes the stimulus imparted to the reflex system by the necessary manipulation is itself enough to excite contraction, but not always." (*London Lancet*, December, 1857.)

It will be observed that in the foregoing case I acted upon the suggestions of Dr. Barnes, as nearly as the circumstances would permit. The operation failing to excite contraction, the tampon was used without loss of time. This was aided by stimulants, opiates, cold applied to the abdomen and vulva, frictions over the abdominal muscles, injections of cold water into the rectum, and other means, but all failed. Had artificial detachment of that portion of the placenta which must necessarily have separated by the expansion of the cervix been performed before the physiological action of the uterus was annihilated from the loss of blood, or had the exhausting drain been promptly met by the opposing tampon, and the blood compelled to coagulate at the mouths of the vessels from which it flowed, there can be no doubt that efficient contractions would have set up in due time, and the fatal result have been averted.

